# Modeling and Performance Optimization of an Irreversible Two-Stage Combined Thermal Brownian Heat Engine

**DOI:** 10.3390/e23040419

**Published:** 2021-03-31

**Authors:** Congzheng Qi, Zemin Ding, Lingen Chen, Yanlin Ge, Huijun Feng

**Affiliations:** 1 Institute of Thermal Science and Power Engineering, Wuhan Institute of Technology, Wuhan 430205, China; 18571499210@139.com (C.Q.); geyali9@hotmail.com (Y.G.); huijunfeng@139.com (H.F.); 2 School of Mechanical & Electrical Engineering, Wuhan Institute of Technology, Wuhan 430205, China; 3 College of Power Engineering, Naval University of Engineering, Wuhan 430033, China; zeminding@hotmail.com

**Keywords:** finite time thermodynamics, thermal Brownian heat engine, combined cycle, power output, efficiency, performance optimization

## Abstract

Based on finite time thermodynamics, an irreversible combined thermal Brownian heat engine model is established in this paper. The model consists of two thermal Brownian heat engines which are operating in tandem with thermal contact with three heat reservoirs. The rates of heat transfer are finite between the heat engine and the reservoir. Considering the heat leakage and the losses caused by kinetic energy change of particles, the formulas of steady current, power output and efficiency are derived. The power output and efficiency of combined heat engine are smaller than that of single heat engine operating between reservoirs with same temperatures. When the potential filed is free from external load, the effects of asymmetry of the potential, barrier height and heat leakage on the performance of the combined heat engine are analyzed. When the potential field is free from external load, the effects of basic design parameters on the performance of the combined heat engine are analyzed. The optimal power and efficiency are obtained by optimizing the barrier heights of two heat engines. The optimal working regions are obtained. There is optimal temperature ratio which maximize the overall power output or efficiency. When the potential filed is subjected to external load, effect of external load is analyzed. The steady current decreases versus external load; the power output and efficiency are monotonically increasing versus external load.

## 1. Introduction

Similar to macro motor, micro energy conversion devices can transfer energy in micro field, such as Brownian motor and energy selective electron engine. Brownian motor is a micro-nano device that can use micro-scale energy [1,2]. It realizes energy conversion through the movement of microscopic particles in viscous medium (VM) with different reservoirs. Many scholars studied the thermodynamic performance of such micro-motors and drew a lot of meaningful conclusions [3].

Finite time thermodynamics (FTT) theory [4,5,6,7] can consider various losses in actual processes and cycles and obtain more accurate results. In the studies of finite time thermodynamics, there exist two kinds of pivotal problems. The first one is to search the extreme of the optimization objective for a given cycle. Another one is to search the optimal path for a preset objective. FTT theory has been applied for performance optimization of various macro energy systems. The applications of FTT include many aspects and the two major aspects are optimal configurations [8,9,10,11,12,13,14,15,16,17,18,19,20,21,22,23,24] and optimal performances [25,26,27,28,29,30,31,32,33,34,35,36,37,38,39,40,41,42,43,44,45,46,47,48,49,50,51,52,53,54,55,56,57,58,59,60,61,62,63,64,65,66,67,68] studies.

Although the operating principles of micro-nano motors are quite different from those of macro motors, FTT theory is also applicable to the study of micro motors [1]. Thermal Brownian motor is driven by temperature difference. The particles migrate directionally and exchange energy with reservoirs. Research shows that the Brownian motor can work as a heat engine (HE) or refrigerator. Asfaw and Bekele [69] modeled a thermal Brownian heat engine (TBHE) of a particle drifting in potential field. The heat flow expressions of the system were given. They proved that it could also work as refrigerator. Asfaw and Bekele [70] modeled a Brownian motor of a particle motioning in one-dimensional lattice. The regions of the system as HE or refrigerator were determined. Asfaw and Bekele [71] established a Brownian motor model. They obtained the parameters range of the system working as HE or refrigerator. Van den Broeck and Kawai [72] studied a completely solvable Brownian refrigerator model. The heat flow of the numerical calculation was compared with the result of the molecular dynamics simulation. Similarly, the Brownian motor can work as a heat pump. Ding et al. [73] modeled an irreversible thermal Brownian heat pump. The effects of parameters on system were analyzed. The range of basic parameters were obtained. Van den Broek et al. [74] analyzed a chiral Brownian heat pump model. The above scholars studied the performance of Brownian motor under three conditions.

Some scholars noticed that some irreversible losses cannot be ignored. Zhang et al. [75] studied a TBHE with kinetic energy loss. The curves of power and efficiency were loop-shaped. Zhang et al. [76] studied a TBHE model considered kinetic and potential energy loss under external load. They obtained the parameters of the system at maximum power or efficiency. Ai et al. [77,78] analyzed the irreversible TBHE [79] and refrigerator [80] with kinetic energy loss. The optimal characteristics of efficiency and coefficient of performance were obtained. Asfaw [81] researched the effect of serrated potential on TBHE. He found that the thermodynamic performance could be improved by subdividing the sawtooth potential. Asfaw [79] analyzed the effect of thermal inhomogeneity of potential field on TBHE. Some scholars studied Brownian motor by other methods. Gao et al. [80] analyzed the effect of irreversible losses on Onsager coefficients. Gao and Chen [82] established an equivalent TBHE model. They studied it with non-equilibrium thermodynamics theory and obtained the Onsager coefficients at maximum power. The research mentioned above mainly focused on the Brownian motors with two reservoirs. Different optimization objective functions, from power, efficiency to ecological function in single-stage Brownian motor have been studied widely [75,83,84,85,86,87,88,89,90,91,92,93,94,95,96,97,98,99,100,101,102,103,104,105,106]. The system models have been gradually improved and more universal results have been obtained.

For the one-stage HE, the heat in the cold reservoir (CR) is released into the environment directly and results in heat losses. To improve energy utilization efficiency, some scholars try to utilize waste heat by connecting the HEs by using FTT theory. For macro energy systems, Rubin and Andresen [107] modeled a three reservoirs endoreversible combined Carnot HE engine model with one as intermediate heat reservoir. Wu [108] optimized power of combined Carnot HE without intermediate heat reservoir. Chen [109] introduced the irreversibility factor to combined Carnot HE model. Ghasemkhani et al. [65] performed multiobjective optimization of combined Carnot HE. Some scholars [110,111,112,113,114,115,116,117,118] have also studied performance characteristics of combined Carnot HEs with various loss issues and different optimization objectives. For quantum energy systems, Meng et al. [119] modeled a three reservoirs endoreversible combined quantum Carnot HE with one as intermediate heat reservoir and Chen et al. [120] modeled irreversible two-stage combined HE with quantum gases.

## 2. Modeling of Irreversible Combined Thermal Brownian Heat Engine

**Figure 1:**
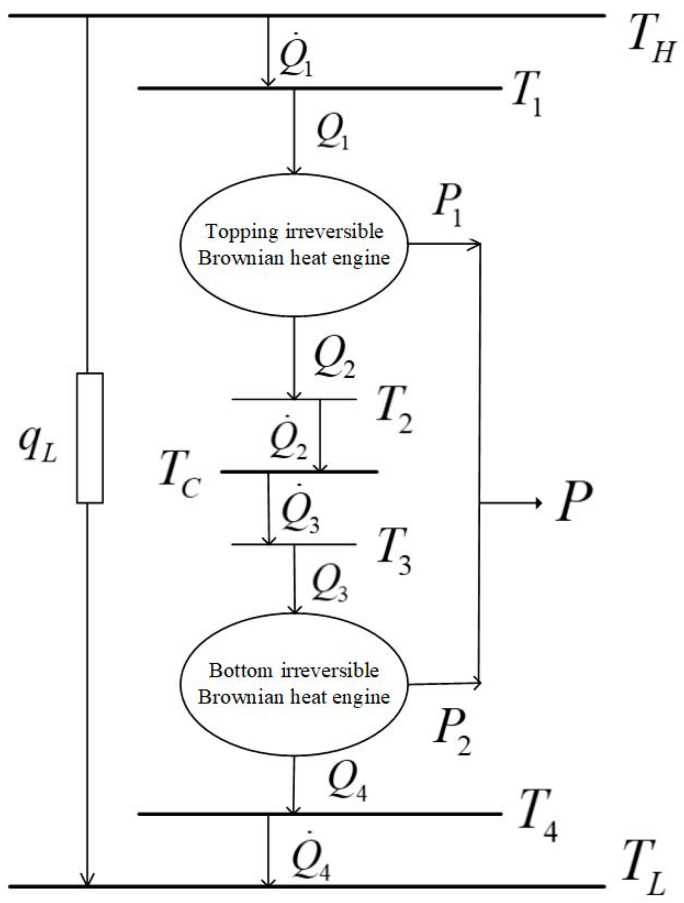
Irreversible combined thermal Brownian heat engine model.

Figure 1 shows an irreversible combined TBHE model working with three reservoirs with the temperatures $T_{H}$, $T_{C}$ and $T_{L}$. The model consists of two irreversibly TBHEs with different barrier height and the two TBHEs are operating in tandem. The bottom TBHE takes the CR of the topping TBHE as hot reservoir (HR). The lower TBHE absorbs heat from the CR of the upper TBHE. The heat dissipated by the upper TBHE is reused by lower TBHE. The intermediate reservoir $T_{C}$ is the CR of the topping TBHE and the HR of the bottom TBHE. $T_{H}$ is the HR of the topping TBHE and $T_{L}$ is the CR of the bottom TBHE. There are thermal resistances between reservoir and VM and heat transfer rate is finite. The operating temperatures of the topping TBHE are $T_{1}$ and $T_{2}$ and those for the bottom TBHE are $T_{3}$ and $T_{4}$.

The combined TBHE model without an intermediate HR is also feasible in theory. When the intermediate HR is not considered, the topping and bottom TBHEs transfer heat directly through viscous medium. As a result, the temperature of CR $T_{2}$ of the topping HE and the HR $T_{3}$ of the bottom HE is dynamic and the corresponding mathematical solution process becomes very complicated. Therefore, this paper introduces an intermediate reservoir to couple the topping and bottom HEs for determining the temperatures $T_{2}$ and $T_{3}$, similar to that in Ref. [107], for macro combined HE, with which the subsequent solution process is greatly simplified.

The method used in this paper can also be used to research the combined TBHE model without the intermediate HR. Similar research work has been performed for macroscopic combined Carnot HE models without the intermediate HR [108,109]. The performance analysis and optimization of combined TBHE without the intermediate HR will be carried out in future work.

**Figure 2:**
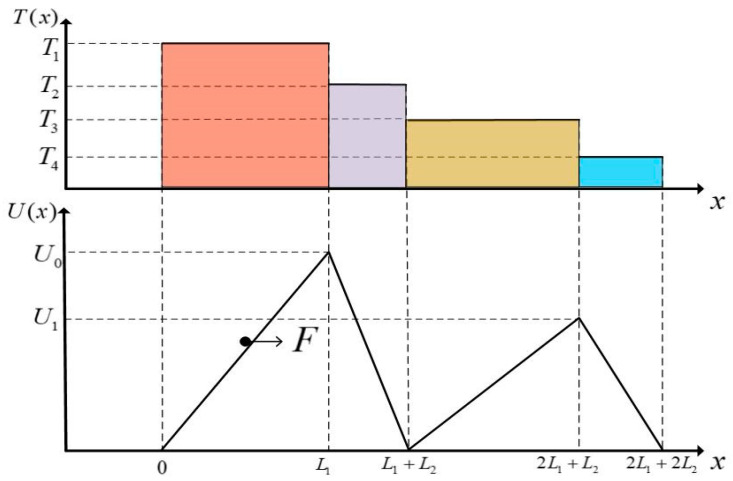
Schematic diagram of combined TBHE.

Figure 2 is the schematic diagram of the combined TBHE. The particle moves in VM with period sawtooth potential field and exchanges heat with the reservoirs through the VM. The VM is an essential part of the present TBHE model without which the Brownian motor cannot move and the entire system cannot operate normally. The potential is electric potential field with different energy heights at different positions. When two TBHEs are operating stably, the model works as a combined TBHE. The model of the micro combined TBHE which can realize multi-stage utilization of energy is established. This research provides a new idea to realize microscopic energy utilization at thermal Brownian motor.

For the topping TBHE, the heat absorption rate is $Q_{1}$; the heat releasing rate is $Q_{2}$; and the power output is $P_{1}$. For the bottom TBHE, the heat absorption rate is $Q_{3}$; the heat releasing rate is $Q_{4}$; and the power output is $P_{2}$. For the combined TBHE, the total power output is P. $q_{L}$ is bypass heat leakage rate between $T_{H}$ and $T_{L}$. It is supposed that the thermal conductivities between the reservoir and the VM are all constant and the temperatures of the reservoirs and VM will not change with time when the system is operating stably. Therefore, $T_{H}>T_{1}>T_{2}>T_{C}>T_{3}>T_{4}>T_{L}$ holds.

The temperature of VM T(x) and the sawtooth potential U(x) are, respectively:

(1) $$T(x)=\left\{ \begin{array}{cc} T_{1}, & 0<x\leq L_{1}\\ T_{2}, & L_{1}<x\leq L_{1}+L_{2}\\ T_{3}, & L_{1}+L_{2}<x\leq 2L_{1}+L_{2}\\ T_{4}, & 2L_{1}+L_{2}<x\leq 2(L_{1}+L_{2}) \end{array}\right.$$

(2) $$U(x)=\left\{ \begin{array}{ll} U_{0}x/L_{1}, & 0<x\leq L_{1}\\ U_{0}(L_{1}/L_{2}-x/L_{2}+1), & L_{1}<x\leq L_{1}+L_{2}\\ U_{1}(x/L_{1}-L_{2}/L_{1}-1), & L_{1}+L_{2}<x\leq 2L_{1}+L_{2}\\ U_{1}(-x/L_{2}+2L_{1}/L_{2}+2), & \;2L_{1}+L_{2}<x\leq 2L_{1}+2L_{2} \end{array}\right.$$

where $L_{1}$ and $L_{2}$ are the lengths of potential field; $U_{0}$ and $U_{1}$ are barrier heights of potential field and the total cycle length is $2L(L=L_{1}+L_{2})$. The sawtooth potential field represents a class of actions which is linearly related to the external load. The barrier height of the potential field is different under different operation conditions. Through the application of a linear sawtooth potential, the mathematical analysis process of the TBHE with inhomogeneous VM can be effectively simplified. Asfaw and Bekele [69] firstly simplified the process of solving the dynamic equation greatly by introducing a sawtooth potential field for the TBHE. The analytical results of Smoluchowski equation make it possible to study the analytical model of a TBHE [69]. In addition, other different kinds of potential fields can be also applied into the study of the TBHE model. Asfaw [81] studied the TBHE with periodic rugged potential field. Asfaw and Bekele [70] and Asfaw [91] studied the TBHE models with discrete ratchet potential. Cheng et al. [121] studied the TBHE with periodic double-barrier sawtooth potential field. This paper studies the TBHE with linear potential field on the bases of previous studies. The application of nonlinear potential field in combined TBHE model will be considered in future research.

When the system works stably, the particle will drift in a directional manner. The heat caused by the friction of Brownian particles through the VM can be expressed as γvL, and γv is friction. The average drift velocity of the particle in a cycle length L (v=JL) is v, and J is the drift velocity of the particle at position z (0<z<L). The expression of J is very complicated; therefore, the self-defined parameters such as G, H and I are introduced to simplify the analytical expression of J. Because of the different barrier heights and operating temperatures of two TBHEs, the steady current is different. The steady current J of upper or lower HE is closely related to the variables such as barrier height U, external load F, the length of potential field L, temperature T, the position of particle z and time t. The analytical expression of J is solved from the Smoluchowski equation for the Brownian particle under specific position z and time t, which makes it possible to analyze the performance of the Brownian motor with a solvable model. According to Refs. [70,75], the steady currents $J_{1}$ and $J_{2}$ of topping and bottom TBHEs are

(3) $$J_{1}=-I_{1}/\left(G_{1}G_{2}+H_{1}I_{1}\right)$$

(4) $$J_{2}=-I_{2}/\left(G_{3}G_{4}+H_{2}I_{2}\right)$$

where the self-defined functions $I_{1}$, $I_{2}$, $H_{1}$, $H_{2}$, $G_{1}$, $G_{2}$, $G_{3}$ and $G_{4}$ are, respectively given by:

(5) $$I_{1}=e^{a-b}-1,I_{2}=e^{c-d}-1$$

(6) $$H_{1}=A_{1}+B_{1}+C_{1},\;H_{2}=A_{2}+B_{2}+C_{2}$$

(7) $$G_{1}=(L_{1}/aT_{1})(1-e^{-a})+(e^{-a}L_{2}/bT_{2})(e^{b}-1),\;G_{2}=(\gamma L_{1}/a)(e^{a}-1)+(\gamma e^{a}L_{2}/b)(1-e^{-b})$$

(8) $$G_{3}=(L_{1}/cT_{3})(1-e^{-c})+(e^{-c}L_{2}/bT_{4})(e^{d}-1),\;G_{4}=(\gamma L_{1}/c)(e^{c}-1)+(\gamma e^{c}L_{2}/d)(1-e^{-d})$$

where γ is the friction coefficient of particles and the self-defined functions $A_{1}$, $B_{1}$, $C_{1}$, $A_{2}$, $B_{2}$ and $C_{2}$ are, respectively:

(9) $$A_{1}=(\gamma L_{1}^{2}/a^{2}T_{1})(e^{-a}+a-1),\;B_{1}=(\gamma L_{1}L_{2}/abT_{2})(1-e^{-a})(e^{b}-1),\;C_{1}=(\gamma L_{2}^{2}/b^{2}T_{2})(e^{b}-b-1)$$

(10) $$A_{2}=(\gamma L_{1}^{2}/c^{2}T_{3})(e^{-c}+c-1),\;B_{2}=(\gamma L_{1}L_{2}/cdT_{4})(1-e^{-c})(e^{d}-1),\;C_{2}=(\gamma L_{2}^{2}/d^{2}T_{4})(e^{d}-d-1)$$

(11) $$a=\left(U_{0}+FL_{1}\right)/T_{1},\;b=\left(U_{0}-FL_{2}\right)/T_{2}$$

(12) $$c=\left(U_{1}+FL_{1}\right)/T_{3},\;d=\left(U_{1}-FL_{2}\right)/T_{4}$$

where F is external load. The drift velocity v of particles is v=JL.

There are three kinds of heat flows for the TBHE. The first is the heat transfer rate when a particle passes through the potential field. The particle absorbs energy to overcome external load F and viscous forces γv. The heat absorption and releasing rates of the topping and bottom TBHEs can be expressed as

(13) $$Q_{1}=U_{0}+(F+\gamma v_{1})L_{1}$$

(14) $$Q_{2}=U_{0}-(F+\gamma v_{1})L_{2}$$

(15) $$Q_{3}=U_{1}+(F+\gamma v_{2})L_{1}$$

(16) $$Q_{4}=U_{1}-(F+\gamma v_{2})L_{2}$$

The second is the energy due to kinetic energy change when a particle moves. The heat flows can be expressed as [122,123]

(17) $$q_{kin1}=k_{B}(T_{1}-T_{2})/2$$

(18) $$q_{kin2}=k_{B}(T_{3}-T_{4})/2$$

where $k_{B}$ is Boltzmann constant, $q_{kin1}$ and $q_{kin2}$ are the energy losses of topping and bottom TBHEs, respectively.

The third is the bypass heat leakage rate $q_{L}$ between reservoirs, which can be expressed as [124,125,126].

(19) $$q_{L}=C_{i}(T_{H}-T_{L})$$

where $C_{i}$ is heat leakage coefficient.

## 3. Main Performance Parameters

According to Equations (13)–(19), the total heat absorption rate ${\dot{Q}}_{1}$ and releasing rate ${\dot{Q}}_{2}$ of the topping TBHE are

(20) $${\dot{Q}}_{1}=Q_{1}+q_{kin1}+q_{L}=U_{0}+(F+\gamma v_{1})L_{1}+k_{B}(T_{1}-T_{2})/2+C_{i}(T_{H}-T_{L})$$

(21) $${\dot{Q}}_{2}=Q_{2}+q_{kin1}=U_{0}-(F+\gamma v_{1})L_{2}+k_{B}(T_{1}-T_{2})/2$$

The power output $P_{1}$ is

(22) $$P_{1}={\dot{Q}}_{1}-{\dot{Q}}_{2}=(F+\gamma v_{1})L+C_{i}(T_{H}-T_{L})$$

The total heat absorption rate ${\dot{Q}}_{3}$ and releasing rate ${\dot{Q}}_{4}$ of the bottom TBHE are

(23) $${\dot{Q}}_{3}=Q_{3}+q_{kin2}=U_{1}+(F+\gamma v_{2})L_{1}+k_{B}(T_{3}-T_{4})/2$$

(24) $${\dot{Q}}_{4}=Q_{4}+q_{kin2}+q_{L}=U_{1}-(F+\gamma v_{2})L_{2}+k_{B}(T_{3}-T_{4})/2+C_{i}(T_{H}-T_{L})$$

The power output $P_{2}$ is

(25) $$P_{2}={\dot{Q}}_{3}-{\dot{Q}}_{4}=(F+\gamma v_{2})L-C_{i}(T_{H}-T_{L})$$

The total P and η of the combined TBHE are

(26) $$P=P_{1}+P_{2}=(F+\gamma v_{1})L+(F+\gamma v_{2})L$$

(27) $$\eta =\frac{P}{{\dot{Q}}_{1}}=\frac{(F+\gamma v_{1})L+(F+\gamma v_{2})L}{U_{0}+(F+\gamma v_{1})L_{1}+k_{B}(T_{1}-T_{2})/2+C_{i}(T_{H}-T_{L})}$$

In order to simplify calculation, some dimensionless parameters are defined: the asymmetry of the potential $\mu =L_{1}/L$, the dimensionless barrier height $U=U_{0}/\left(k_{B}T_{H}\right)$, the ratio of barrier height $n=U_{0}/U_{1}$, the dimensionless external load $f=FL/(k_{B}T_{H})$, $\beta =C_{i}/k_{B}$, $\tau =T_{L}/T_{H}$, $\omega =T_{C}/T_{H}$, $k_{1}=T_{L}/T_{4}$, $k_{2}=T_{3}/T_{C}$, $k_{3}=T_{C}/T_{2}$ and $k_{4}=T_{1}/T_{H}$. It is future defined that $a=k_{B}(U+\mu f)/k_{4}$, $b=k_{3}k_{B}[U-(1-\mu )f]/\omega$, $c=k_{B}(U/n+\mu f)/k_{2}\omega$ and $d=k_{1}k_{B}[U/n-(1-\mu )$f]/τ. The dimensionless forms of $J_{1}$, $J_{2}$, P and the efficiency $\eta^{*}$ can be rewritten as follows:

(28) $$J_{1}^{*}=\frac{J_{1}\gamma L^{2}}{k_{B}T_{H}}=\frac{\left(1-e^{a-b}\right)/k_{B}}{x_{0}+(e^{a-b}-1)y_{0}}$$

(29) $$J_{2}^{*}=\frac{J_{2}\gamma L^{2}}{k_{B}T_{H}}=\frac{\left(1-e^{c-d}\right)/k_{B}}{x_{1}+(e^{c-d}-1)y_{1}}$$

(30) $$P^{*}=\frac{P}{k_{B}T_{H}}=2f+\frac{\left(1-e^{a-b}\right)/k_{B}}{x_{0}+(e^{a-b}-1)y_{0}}+\frac{\left(1-e^{c-d}\right)/k_{B}}{x_{1}+(e^{c-d}-1)y_{1}}$$

(31) $$\eta^{*}=\eta =\frac{2f+\left[\left(1-e^{a-b}\right)/k_{B}\right]/\left[x_{0}+(e^{a-b}-1)y_{0}\right]+\left[\left(1-e^{c-d}\right)/k_{B}\right]/\left[x_{1}+(e^{c-d}-1)y_{1}\right]}{U+\mu f+\left[\mu (1-e^{a-b})/k_{B}\right]/\left[x_{0}+(e^{a-b}-1)y_{0}]+\left(k_{4}-\omega /k_{3}\right)/2+\beta (1-\tau \right)}$$

The self-defined functions $x_{0}$, $y_{0}$, $x_{1}$ and $y_{1}$ are introduced to simplify the writing processes. These functions are defined as, respectively:

(32) $$x_{0}=[\frac{\mu (1-e^{-a})}{ak_{4}}+\frac{k_{3}e^{-a}(1-\mu )(e^{b}-1)}{b\omega }][\frac{\mu (e^{a}-1)}{a}+\frac{e^{a}(1-\mu )(1-e^{-b})}{b}]$$

(33) $$y_{0}=\frac{\mu^{2}(e^{-a}+a-1)}{a^{2}k_{4}}+\frac{\mu k_{3}(1-\mu )(1-e^{-a})(e^{b}-1)}{ab\omega }+\frac{k_{3}{\left(1-\mu \right)}^{2}(e^{b}-b-1)}{b^{2}\omega }$$

(34) $$x_{1}=[\frac{\mu (1-e^{-c})}{ck_{2}\omega }+\frac{k_{1}e^{-c}(1-\mu )(e^{d}-1)}{d\tau }][\frac{\mu (e^{c}-1)}{c}+\frac{e^{c}(1-\mu )(1-e^{-d})}{d}]$$

(35) $$y_{1}=\frac{\mu^{2}(e^{-c}+c-1)}{c^{2}k_{2}\omega }+\frac{\mu (1-\mu )k_{1}(1-e^{-c})(e^{d}-1)}{cd\tau }+\frac{k_{1}{\left(1-\mu \right)}^{2}(e^{d}-d-1)}{d^{2}\tau }$$

## 4. Optimal Performance Characteristics without External Load

### 4.1. Performance Analysis without External Load

When the combined TBHE is free from external load (f=0), the analytical expressions of power ($P^{*}$) and efficiency ($\eta^{*}$) are

(36) $$P^{*}=\frac{Pt}{k_{B}T_{H}}=\frac{(1-e^{a-b})\;t/k_{B}}{x_{0}+(e^{a-b}-1)y_{0}}+\frac{(1-e^{c-d})\;t/k_{B}}{x_{1}+(e^{c-d}-1)y_{1}}$$

(37) $$\eta^{*}=\frac{\left[\left(1-e^{a-b}\right)/k_{B}\right]/\left[x_{0}+(e^{a-b}-1)y_{0}\right]+\left[\left(1-e^{c-d}\right)/k_{B}\right]/\left[x_{1}+(e^{c-d}-1)y_{1}\right]}{U+\left[\mu (1-e^{a-b})/k_{B}\right]/\left[x_{0}+(e^{a-b}-1)y_{0}]+\left(k_{4}-\omega /k_{3}\right)/2+\beta (1-\tau \right)}$$

where $a=k_{B}U/k_{4}$, $b=k_{3}k_{B}U/\omega$, $c=k_{B}U/nk_{2}\omega$ and $d=k_{1}k_{B}U/n\tau$.

The performance parameters $P^{*}$ and $\eta^{*}$ are the functions of variables such as U, n, μ, $k_{i}(i=1,2,3,4)$, ω and τ. When the temperature of reservoirs $T_{H}$ and $T_{L}$ are fixed, the temperature ratio $\tau =T_{L}/T_{H}$ is fixed. The temperature of intermediate reservoir is $T_{C}$. The parameter ω ($\omega =T_{C}/T_{H}$) is defined as the temperature ratio of intermediate reservoir to the HR. Due to $T_{L}<T_{C}<T_{H}$, one has 0<τ<ω<1. The dimensionless barrier height U ($U=U_{0}/\left(k_{B}T_{H}\right)$) is taken as a major control variable of the system. The ratio of barrier height is defined as $n=U_{0}/U_{1}$ and one has n>0. The asymmetry of the potential is defined as $\mu =L_{1}/L$ and one has 0<μ<1. These variables will affect the performance directly. Therefore, it is necessary to analyze their effects emphatically. The performance of the combined TBHE without external load is analyzed by numerical calculation. To simplify the analyses, it is supposed that the thermal conductivities are equal between reservoir and vicious medium and $k=k_{1}=k_{2}=k_{3}=k_{4}$. The combined TBHE is operating between reservoirs with fixed temperature $T_{H}$ and $T_{L}$, and τ is fixed. 

**Figure 3:**
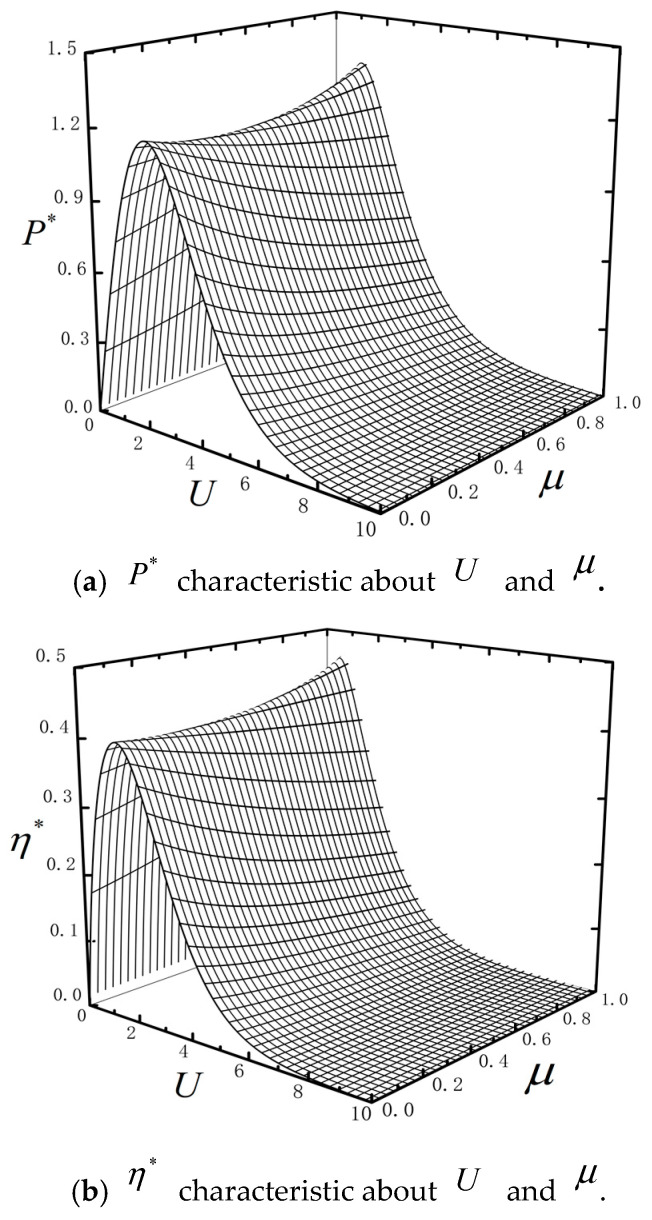
$P^{*}$ and $\eta^{*}$ characteristics about U and μ.

Figure 3 shows $P^{*}$ and $\eta^{*}$ characteristics about U and μ with τ=0.1, ω=0.4, k=0.95, n=2 and *C_i_* = 2. The $P^{*}$ and $\eta^{*}$ performance characteristics are similar. When μ is fixed, $P^{*}$ and $\eta^{*}$ have maximums about U. This is because the barrier height affects the motion and the heat exchange of particle. The steady current increases with the increase of barrier height and the $P^{*}$ and $\eta^{*}$ increase; as the barrier height gets higher and higher, the steady current decreases but the absorbed rate increases from HR $T_{H}$ and the $P^{*}$ and $\eta^{*}$ decrease. when U is fixed, $P^{*}$ and $\eta^{*}$ are monotonous about μ. The maximum power ($P_{\max}^{*}$) and maximum efficiency ($\eta_{\max}^{*}$) decrease first and then increase versus μ. The heat absorption of the particle for overcoming friction is influenced by the parameter μ.

**Figure 4:**
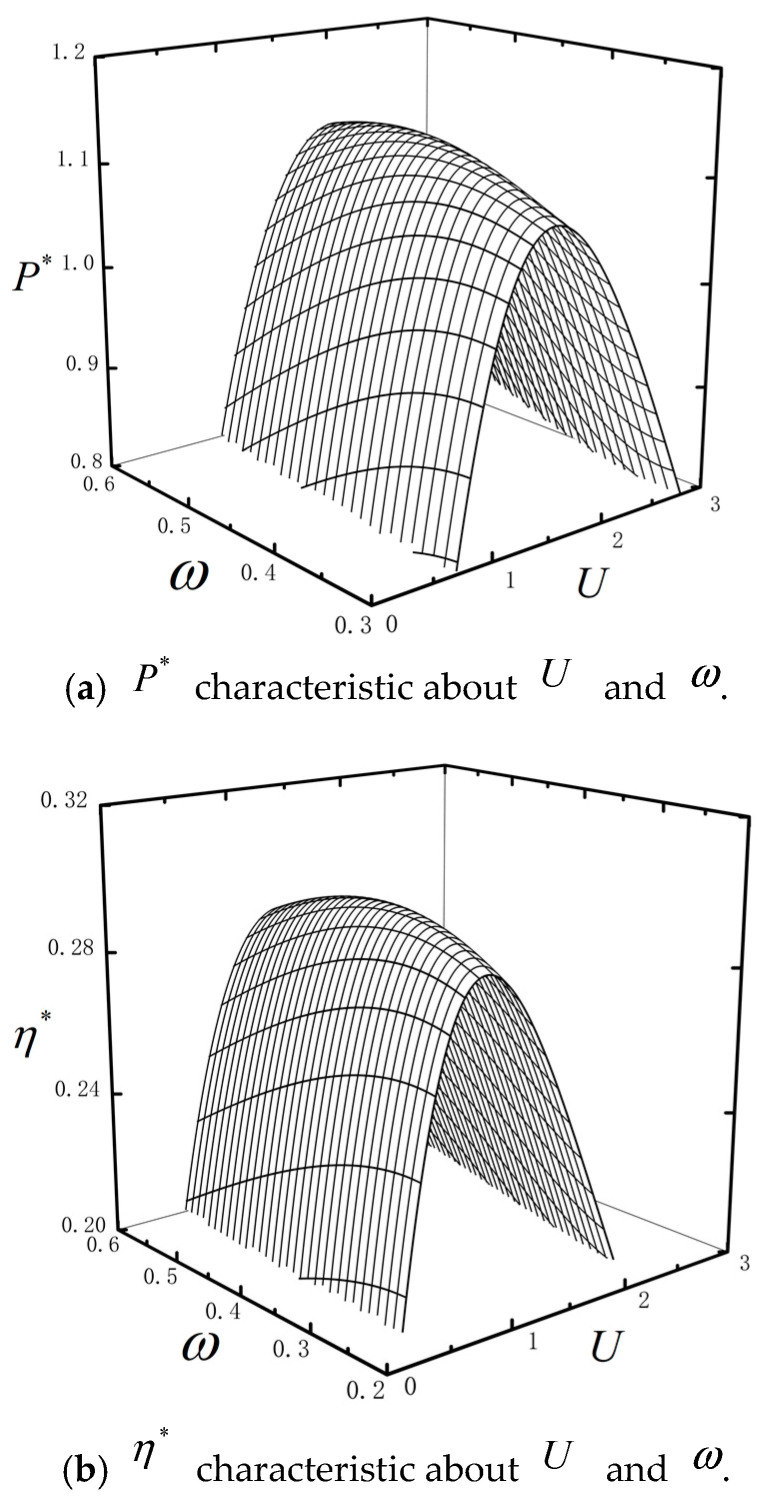
$P^{*}$ and $\eta^{*}$ characteristics about U and ω.

Figure 4 show $P^{*}$ and $\eta^{*}$ characteristics about U and ω with τ=0.1, μ=0.4, k=0.95, n=2 and *C_i_* = 2. It is shown that the performance of the combined TBHE is closely related to the temperature ratio ω. When τ and U are fixed, $P^{*}$ and $\eta^{*}$ have peaks about ω. That means that when $T_{H}$ and $T_{L}$ are fixed, there is an optimal temperature $T_{C}$ for the intermediate reservoir to maximize $P^{*}$ or $\eta^{*}$. Figure 3 and Figure 4 research the characteristics of the $P^{*}$ and $\eta^{*}$ about μ or ω with different parameters. Although the shapes of the figures are similar, the values of the corresponding parameters are different when the power and efficiency are maximized. This section studies the performance characteristics of the system operating at maximum power or efficiency and explains the operating principle. Therefore, Figure 3 and Figure 4 show the above shapes.

**Figure 5:**
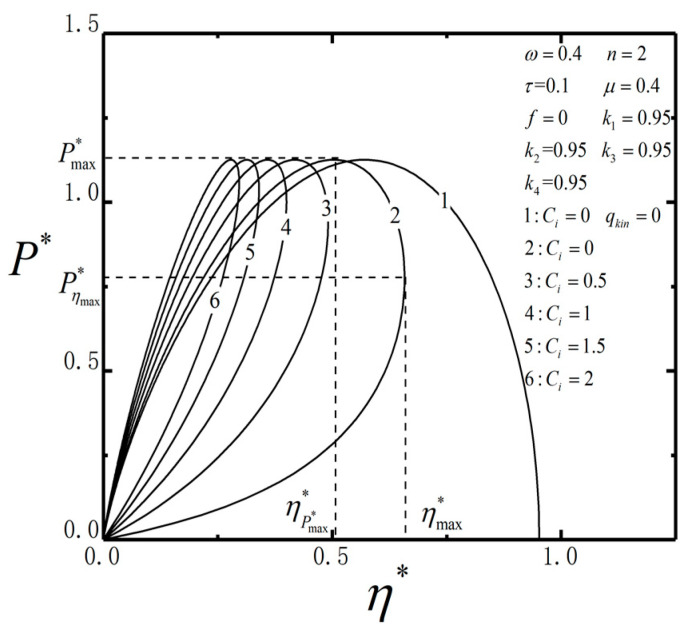
The curves of $P^{*}-\eta^{*}$ about $C_{i}$.

Figure 5 shows the curves of $P^{*}-$$\eta^{*}$ about $C_{i}$. The curve is parabolic one if $q_{L}$ and $q_{kin}$ are ignored as shown by curve 1; and it is loop-shaped one if $q_{L}$ and $q_{kin}$ are considered. The effects of heat leakage and the heat flow due to the change of kinetic energy on the system performance are similar to the effect of heat leakage on the macro thermodynamic systems. Therefore, the curve of power output and efficiency presents a loop-shaped characteristic similar to macro heat engine. When $C_{i}$ increases, the area of the loop-shaped curve decreases; the power $P^{*}{}_{\max}$ remains constant whereas the efficiency $\eta_{\max}$ decreases. The optimal working regions are $P_{\eta^{*}{}_{\max}}^{*}<P^{*}<P_{\max}^{*}$ and $\eta^{*}{}_{P^{*}{}_{\max}}<\eta^{*}<\eta^{*}{}_{\max}$, where both $P^{*}$ and $\eta^{*}$ can be maintained as large.

**Figure 6:**
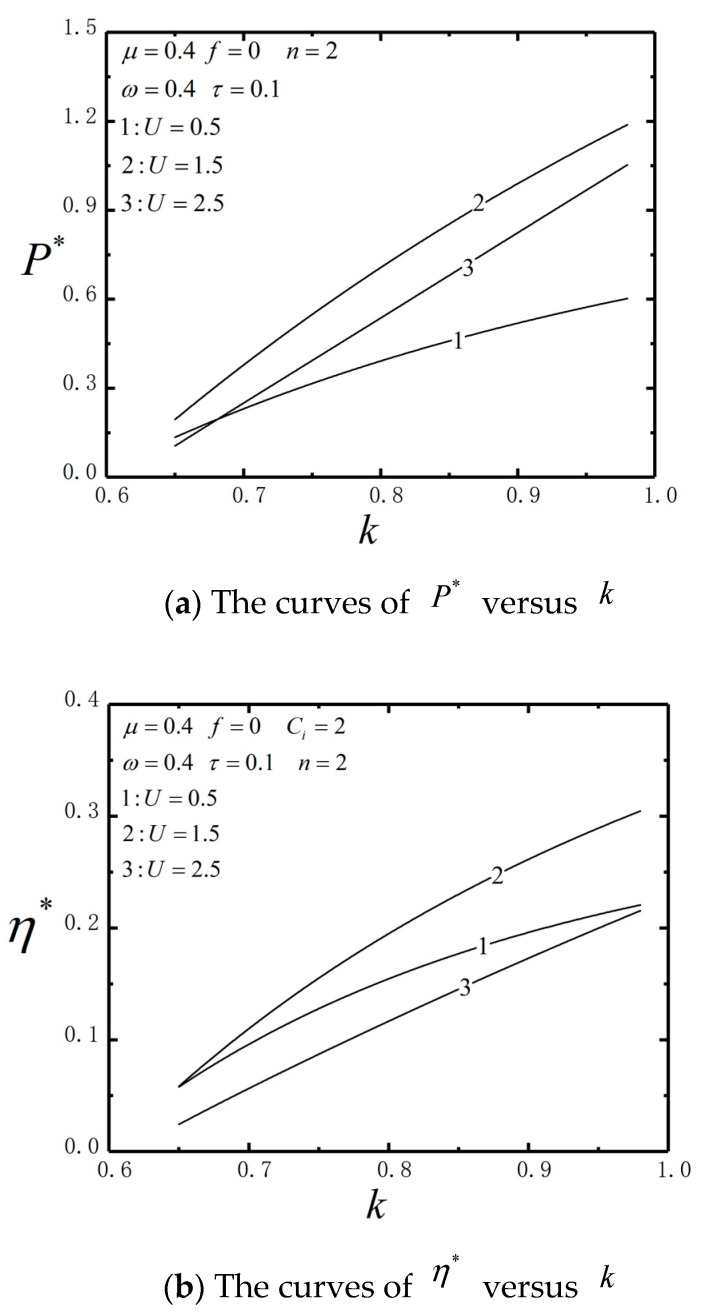
$P^{*}$ and $\eta^{*}$ characteristics versus k.

Figure 6 shows the characteristics of $P^{*}$ and $\eta^{*}$ versus k with τ=0.1, μ=0.4 , n=2, ω=0.4 and $C_{i}=2$. $P^{*}$ and $\eta^{*}$ monotonically increase about k. It indicates that the higher the thermal conductivities between the vicious medium and reservoir, the better $P^{*}$ and $\eta^{*}$ of the combined TBHE. Enhancing the heat transfer between reservoir and TBHE can improve $P^{*}$ and $\eta^{*}$ of the system.

**Figure 7:**
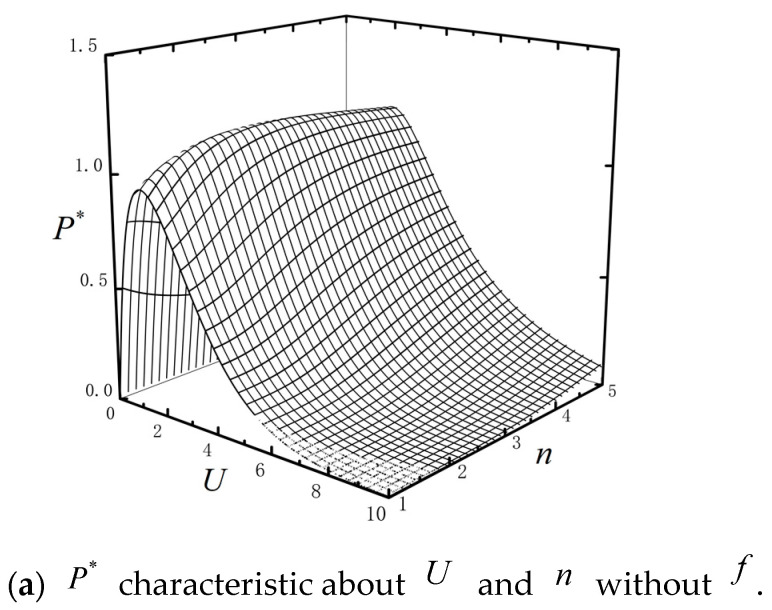

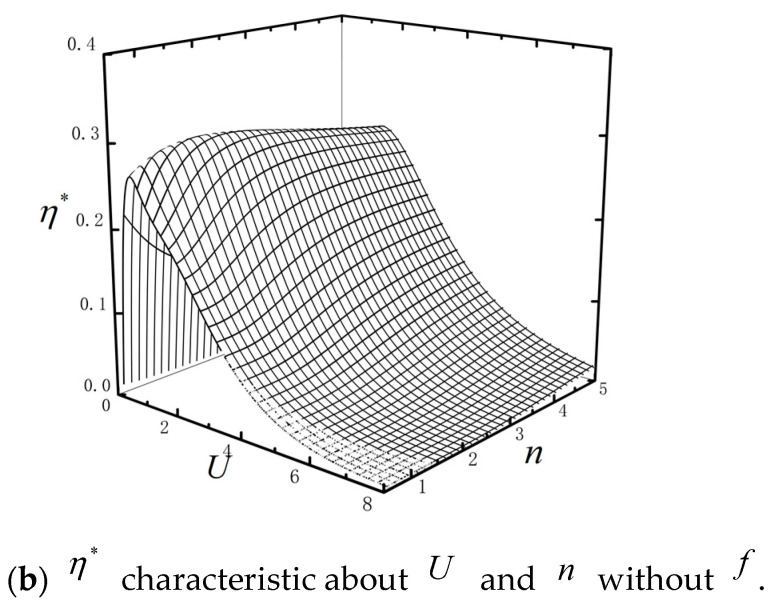
$P^{*}$ and $\eta^{*}$ characteristics about U and n without f.

According to above analyses, one can find that the barrier height U has obvious impact on the system performance. When the upper heat engine works normally, the performance of lower heat engine can be optimized by adjusting the barrier height of the lower engine and the performance of the combined system can be optimized. Therefore, the relationship between barrier heights $U_{0}$ and $U_{1}$ of the two-stage combined TBHE is studied in detail. Figure 7 shows the $P^{*}$ and $\eta^{*}$ characteristics about U and n without external load with τ=0.1, $C_{i}=2$, ω=0.4, μ=0.4 and k=0.95. It can be seen that $P^{*}$ and $\eta^{*}$ have peaks about U and n. There are appropriate U and n which maximize $P^{*}$ or $\eta^{*}$, respectively. When the topping HE operating is stable, the barrier height of bottom HE can be adjusted to make the combined TBHE operating at optimal performance. It is of great significance for the analysis and parameter design of combined TBHE.

### 4.2. Optimal Performance without External Load

When ${\left(\partial P^{*}/\partial U\right)}_{n}=0$ and ${\left(\partial P^{*}/\partial n\right)}_{U}=0$, according to Equations (36) and (37), $P^{*}{}_{\max}$ and the corresponding efficiency ($\eta^{*}{}_{P*}$) can be obtained. Similarly, when $\eta^{*}$ is maximized, $\eta_{\max}^{*}$ and the corresponding power ($P^{*}{}_{\eta^{*}}$) can be obtained by the method of evaluation extreme value. The performance analysis and optimization work performed for the combined two-stage thermal Brownian heat engine belongs to the first type of research problem of finite time thermodynamics. Due to its special working mechanism, the expressions of performance parameters such as power and efficiency respect to its design parameters are highly non-linear. It is very difficult to obtain the analytical solution. Therefore, numerical methods are applied for intensive analyses.

**Figure 8:**
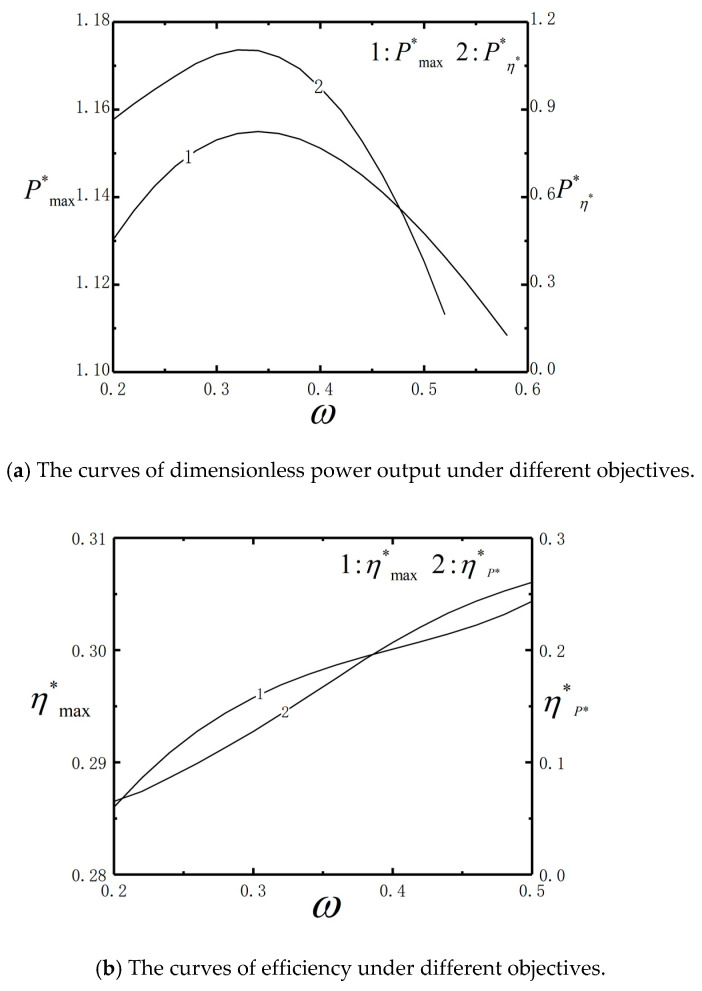
The curves of dimensionless power output and efficiency under different objectives.

Figure 8 shows the performance of dimensionless power output ($P_{\max}^{*}$,$P_{\eta^{*}}^{*}$) and efficiency ($\eta_{\max}^{*}$, $\eta_{P^{*}}^{*}$) versus ω with the optimal barrier height. The variation ranges of $P_{\max}^{*}$ and $\eta_{\max}^{*}$ are small, but the variation ranges of $P_{\eta^{*}}^{*}$ and $\eta_{P^{*}}^{*}$ are large. After optimizing the barrier heights $U_{0}$ and n, $\eta_{\max}^{*}$ is monotonically increasing versus ω. This is different from the results shown by Figure 5, where the $\eta_{\max}^{*}$ increases firstly, and then decreases. The $P_{\max}^{*}$ and $\eta_{\max}^{*}$ cannot be satisfied simultaneously. Different optimization objectives can be chosen under different design requirement.

## 5. The Performance Characteristics with External Load

The barrier height will change when the potential field is influenced by external load, which will affect the movement of the particle. Therefore, the performance of the system needs to be further analyzed.

**Figure 9:**
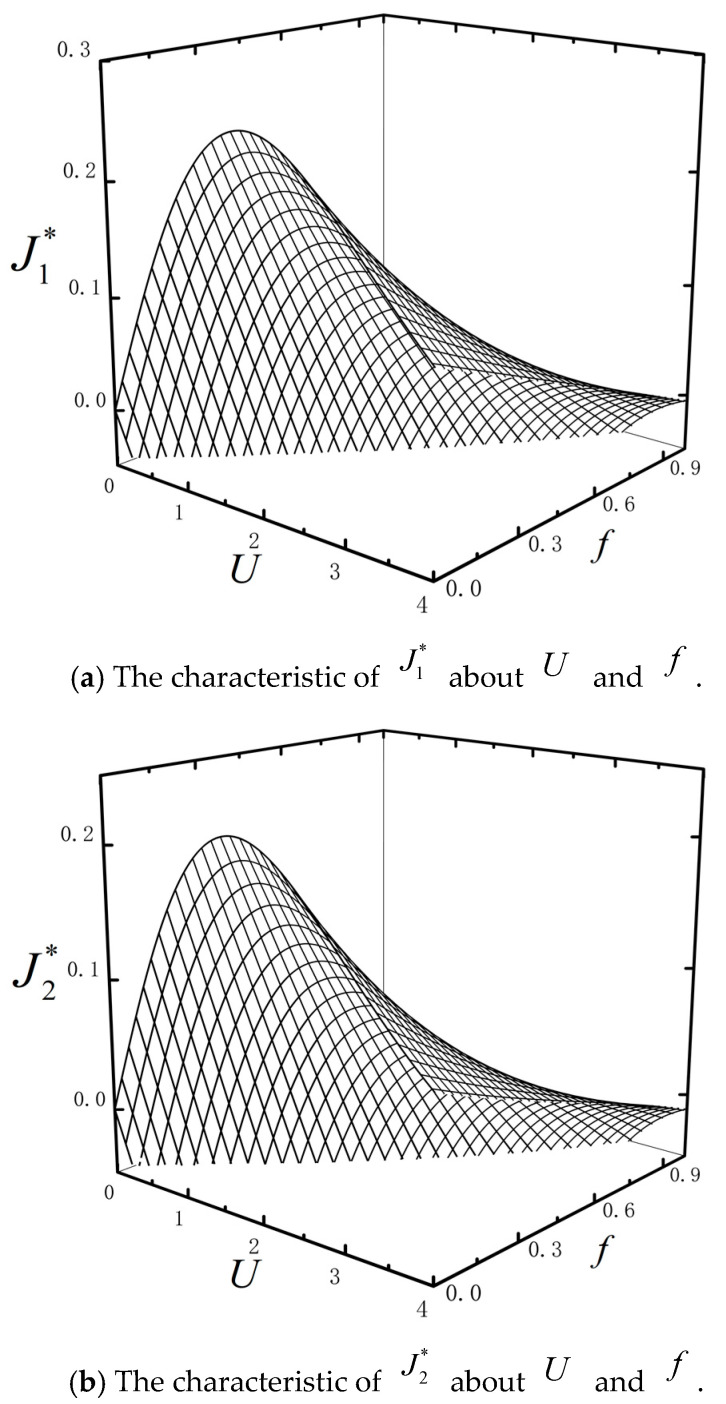
The characteristics of $J_{1}^{*}$ and $J_{2}^{*}$ about U and f.

Figure 9 shows the characteristics $J_{1}^{*}$ and $J_{2}^{*}$ about U and f with ω=0.4, τ=0.2, μ=0.4, k=0.8, n=2 and $C_{i}=2$. The steady current decreases monotonically with external load. This indicates that the velocity of the particles decreases when the sawtooth potential is acted upon by external load. When external load f increases further, $J_{1}^{*}$ or $J_{2}^{*}$ is less than zero. This means that the particle moves in the opposite direction and the Brownian motor works as a refrigerator. If the combined TBHE works normally, the particles are moving to the right for the topping and bottom TBHEs. The external load f should satisfy $J_{1}^{*}>0$ and $J_{2}^{*}>0$.

**Figure 10:**
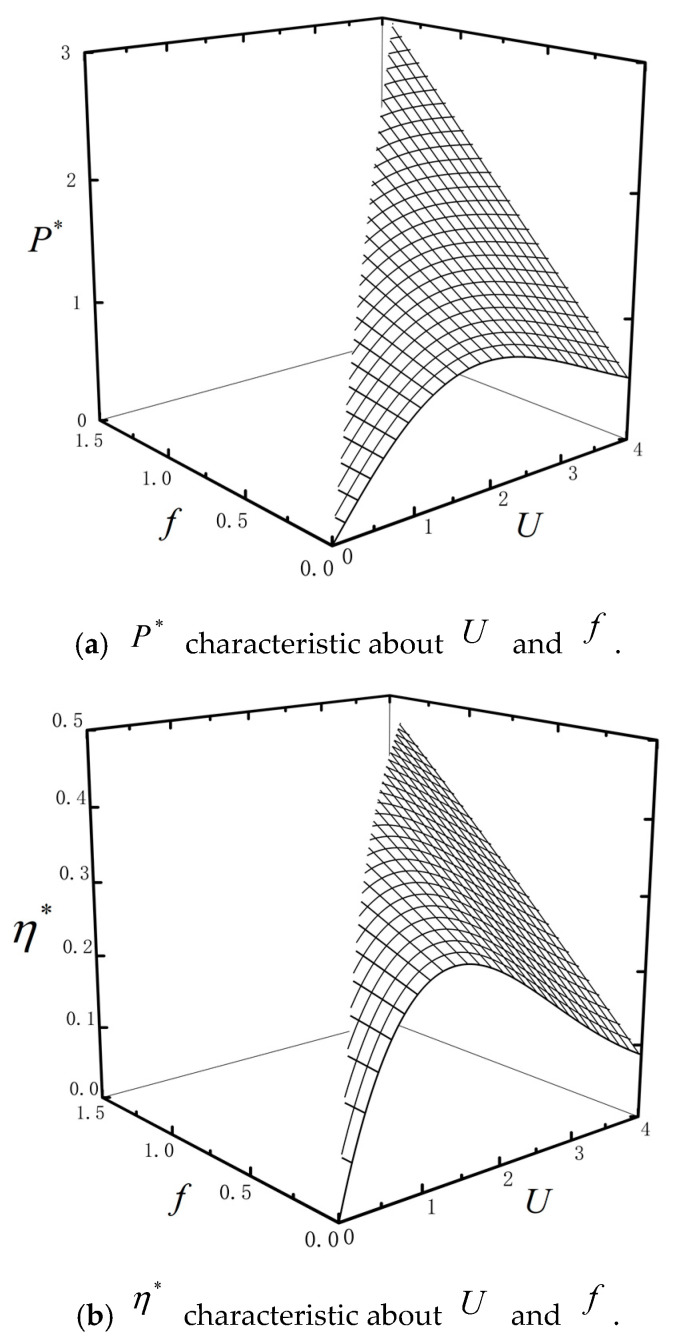
$P^{*}$ and $\eta^{*}$ characteristics about U and f.

**Figure 11:**
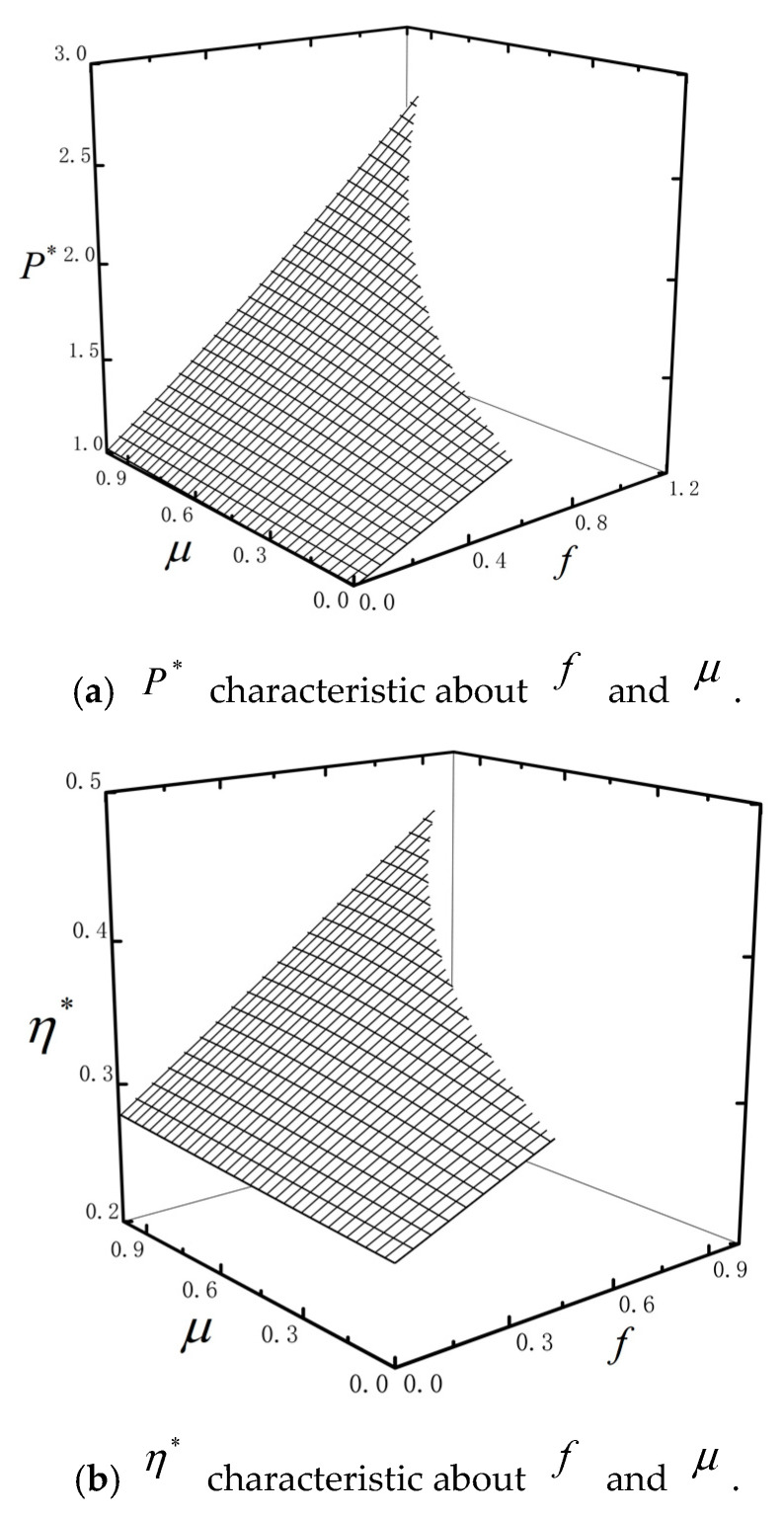
$P^{*}$ and $\eta^{*}$ characteristics about f and μ.

Figure 10 shows the characteristics of $P^{*}$ and $\eta^{*}$ about U and f with ω=0.4, τ=0.2, μ=0.4, k=0.8, n=2 and $C_{i}=2$. Figure 11 shows the characteristics of $P^{*}$ and $\eta^{*}$ about f and μ with ω=0.4, τ=0.2, U=2, k=0.8, n=2 and $C_{i}=2$. $P^{*}$ and $\eta^{*}$ are monotonically increasing versus f. The reason is that the particle needs to absorb heat in order to overcome external load. $P^{*}$ and $\eta^{*}$ increase first and then decreasing versus U when f is small; but $P^{*}$ and $\eta^{*}$ are monotonically decreasing versus U when f is large. The surfaces are truncated as shown by Figure 10. This is because when the barrier height $U_{0}$ and $U_{1}$ are fixed, the external loaf f can’t be too large if the combined TBHE is to work normally. When f is fixed, μ has little impact on $P^{*}$ and $\eta^{*}$. However, $P_{\max}^{*}$ and $\eta_{\max}^{*}$ are monotonically increasing versus μ as shown by Figure 11. The heat absorption of the particle for overcoming external load is influenced by the parameter μ.

**Figure 12:**
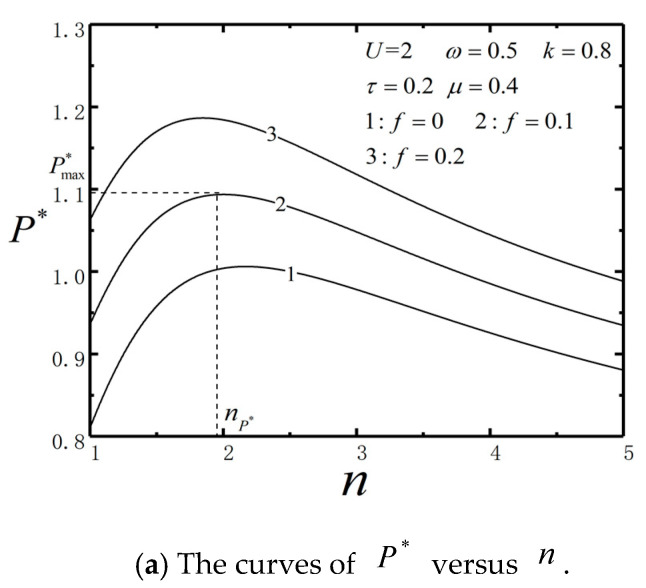

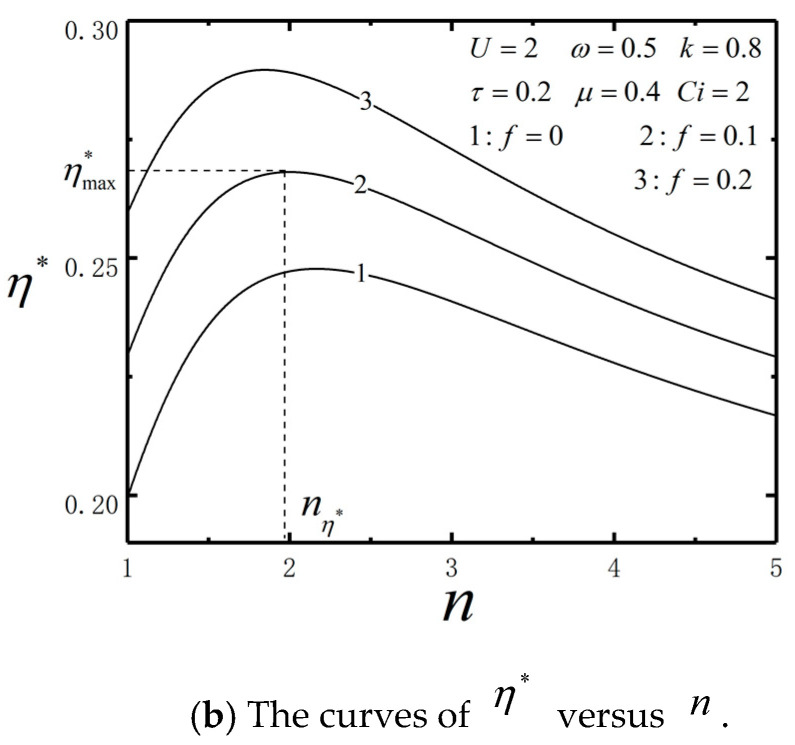
$P^{*}$ and $\eta^{*}$ characteristics versus n.

Figure 12 shows the characteristics of $P^{*}$ and $\eta^{*}$ versus n with different f. External load makes $P^{*}$ and $\eta^{*}$ increased significantly. When U is fixed, there is an optimal $n_{P^{*}}$ (or $n_{\eta^{*}}$) to maximize $P^{*}$ (or $\eta^{*}$). When the upper HE works normally, the performance of lower HE can be optimized by adjusting the barrier height of the lower engine and the performance of the combined TBHE can be improved. If the ratio of barrier height is set properly, $P^{*}$ or $\eta^{*}$ will increase. These characteristics are consistent with Figure 7.

## 6. Performance Comparison with Single Cycle

The combined TBHE can improve power output and efficiency effectively by expanding the temperature range. In order to explore the differences between the micro and macro combined HEs, this section compares the performance characteristics with macro combined HE. For the irreversible single TBHE operating between reservoirs $T_{H}$ and $T_{L}$, it is supposed that the temperatures of VM remains at $T_{1}$ and $T_{4}$, the power output $P_{1}^{*}$ and efficiency $\eta_{1}^{*}$ are [75]:

(38) $$P_{3}^{*}=f+\frac{\left(1-e^{a_{1}-b_{1}}\right)/k_{B}}{x_{2}+(e^{a_{1}-b_{1}}-1)y_{2}}$$

(39) $$\eta_{3}^{*}=\frac{f+\left[\left(1-e^{a_{1}-b_{1}}\right)/k_{B}\right]/\left[x_{2}+(e^{a_{1}-b_{1}}-1)y_{2}\right]}{U+\mu f+\left[\mu (1-e^{a_{1}-b_{1}})/k_{B}\right]/\left[x_{2}+(e^{a_{1}-b_{1}}-1)y_{2}]+\left(k_{4}-\tau /k_{1}\right)/2+\beta (1-\tau \right)}$$

where the self-defined functions $a_{1}$, $b_{1}$, $x_{2}$ and $y_{2}$ are, respectively:

(40) $$a_{1}=k_{B}(U+\mu f)/k_{4},\;b_{1}=k_{1}k_{B}[U-(1-\mu )f]/\tau$$

(41) $$x_{2}=[\frac{\mu (1-e^{-a_{1}})}{a_{1}k_{4}}+\frac{k_{1}e^{-a_{1}}(1-\mu )(e^{b_{1}}-1)}{b_{1}\tau }][\frac{\mu (e^{a_{1}}-1)}{a_{1}}+\frac{e^{a_{1}}(1-\mu )(1-e^{-b_{1}})}{b_{1}}]$$

(42) $$y_{2}=\frac{\mu^{2}(e^{-a_{1}}+a_{1}-1)}{a_{1}{}^{2}k_{4}}+\frac{\mu k_{1}(1-\mu )(1-e^{-a_{1}})(e^{b_{1}}-1)}{a_{1}b_{1}\tau }+\frac{k_{1}{\left(1-\mu \right)}^{2}(e^{b_{1}}-b_{1}-1)}{b_{1}{}^{2}\tau }$$

**Figure 13:**
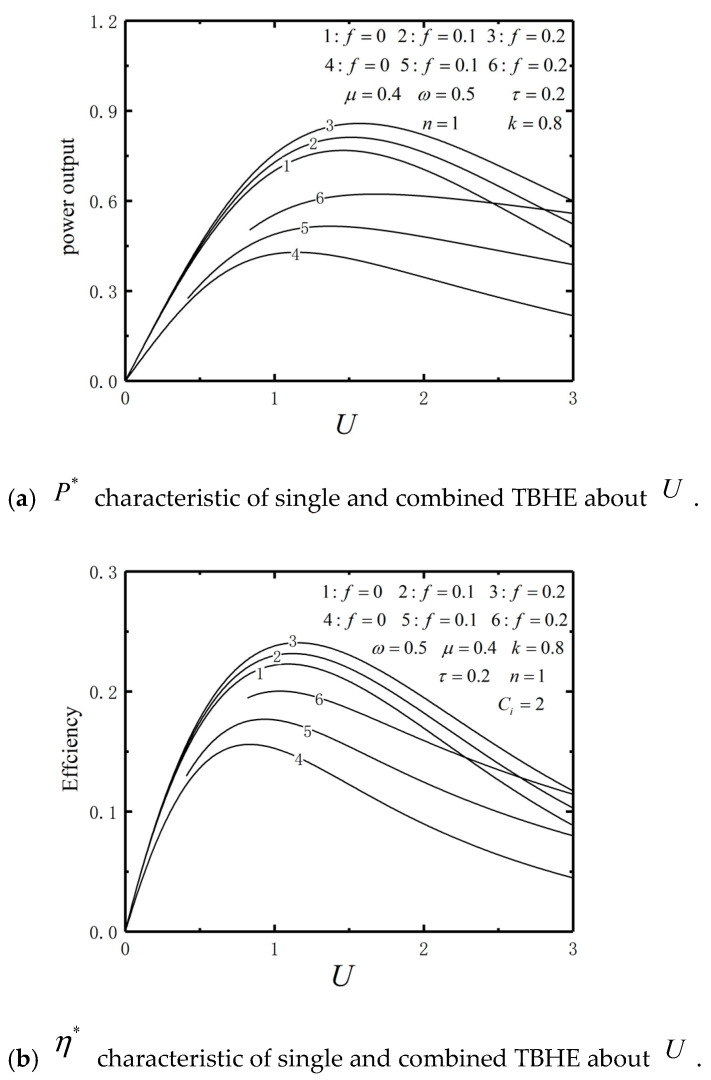
$P^{*}$ and $\eta^{*}$ characteristics of single and combined TBHE about U.

The performance comparison is illustrated by numerical calculation. The parameters are set as ω=0.5, τ=0.2, μ=0.4, k=0.8, n=1 and $C_{i}=2$. Figure 13 shows the $P^{*}$ and $\eta^{*}$ versus U curves of the irreversible single TBHE (curves 1–3) and irreversible combined TBHE (curves 4–6), respectively. Table 1 and Table 2 list the power output and efficiency performance comparison between single and combined TBHE with U=1.5. It shows that the values of $P^{*}$ and $\eta^{*}$ for the irreversible single TBHE are higher than those of the combined TBHE. That is because there exist thermal resistances between the reservoir and the VM. The results about power output are consistent with the analyses of Refs. [107,108,109] for conventional macroscopic energy conversion systems, but the reasons are different. The power of macro heat engine decreases due to the total thermal resistance increases, there are three reasons for the power reduction of the combined TBHE:

1. The irreversible combined TBHE is connected by intermediate reservoir, which leads to the increased overall heat resistance increase.
2. The TBHE exchange energy through particle moves, which is different from macro heat engine. The energy releasing from upper heat engine can’t be absorbed completely by lower heat engine and as a result the efficiency decreases.
3. Because the intermediate reservoir is considered, the temperature of the reservoirs of upper heat engine changes and the velocity of particle motion changes. The upper heat engine absorbs less energy from the hot reservoir $T_{H}$. As a result, the total power output decreases.

It is supposed that the single or combined HE is operating between two heat reservoirs with fixed temperatures. For the one-stage TBHE, there are two heat transfer processes between two reservoirs. For the two-stage combined TBHE, the bottom HE has two heat transfer processes and there are four heat transfer processes in total. As a result, the overall heat resistance increases and the power and efficiency decrease. For the three-stage combined TBHE, two heat transfer processes are added, there are six heat transfer processes. As a result, the power and efficiency decrease continually. Therefore, as the number of HEs operating in tandem increases, the overall heat resistance increases and the power and efficiency decrease. Some scholars have studied the macro combined Carnot HE model and systematically expounded the effect of heat resistance on the performance of multi-stage combined Carnot HE [99,109].

One can find from the present analyses that, when the bottom TBHE is placed below the topping TBHE, the entire power output and efficiency can be greatly improved due to the expended working temperature of the entire system. The results can be applied to the performance optimization of the combined energy conversion systems with multiple HRs.

## 7. Conclusions

In this paper, an irreversible combined thermal Brownian heat engine model is established by FTT theory. The effects of basic parameters on the performance of the combined thermal Brownian heat engine are analyzed with or without external load. The optimal thermodynamic performance of the combined thermal Brownian heat engine is further studied. The main conclusions are:

1. When the temperature ratio τ is fixed, there is a specific optimal temperature ratio ω which maximize power or efficiency. Reducing the heat leakage coefficient and enhancing the heat transfer between the thermal Brownian heat engines and reservoir can improve the performance. There are suitable barrier height and the ratio of barrier height to make the combined thermal Brownian heat engine work at optimum power and efficiency whether the potential field is affected by the external load or not.
2. When the potential field is free from external load, considering heat leakage and kinetic energy loss, the curves of $P^{*}-\eta$ are loop-shaped ones and the optimal working regions are $P_{\eta^{*}{}_{\max}}^{*}<P^{*}<P_{\max}^{*}$ and $\eta^{*}{}_{P^{*}{}_{\max}}<\eta^{*}<\eta^{*}{}_{\max}$. The maximum power and efficiency decrease first and then increase versus asymmetry of the potential.
3. When the potential field is influenced by external load, the steady current decreases with the increase of external load. The maximum power and efficiency monotonically increase versus the asymmetry of the potential. The power and efficiency are monotonically increasing versus external load.
4. The overall heat resistance of combined thermal Brownian heat engine is bigger than that of single thermal Brownian heat engine, the power or efficiency of combined thermal Brownian heat engine are lower than that of single thermal Brownian heat engine.

## Figures and Tables

**Table 1:** The power output performance comparisons between single and combined TBHE with U=1.5.

External Load	f=0	f=0.5	f=1
Single TBHE $P^{*}$	0.768	0.812	0.857
Combined TBHE $P^{*}$	0.408	0.513	0.62
Increasement of $P^{*}$	−46.9%	−36.8%	−27.7%

**Table 2:** The efficiency performance comparisons between single and combined TBHE with U=1.5.

External Load	f=0	f=0.5	f=1
Single TBHE $\eta^{*}$	0.208	0.219	0.23
Combined TBHE $\eta^{*}$	0.122	0.154	0.186
Increasement of $\eta^{*}$	−41.3%	−29.7%	−19.1%

## Nomenclature

A	self-defined function
a	self-defined function
B	self-defined function
b	self-defined function
C	self-defined function
$C_{i}$	heat leakage coefficient (J/K)
c	self-defined function
F	external load
f	dimensionless external load
GH, I	some defined functions
J	steady current
k	temperature ratio
$k_{B}$	Boltzmann constant (J/K)
L	length of potential field (m)
n	the ratio of barrier height
P	power output (W)
Q	heat flow rate (W)
$q_{kin}$	kinetic energy (W)
$q_{L}$	heat leakage rate (W)
T	temperature ( K)
U	barrier height (J)
v	drift velocity of particle
x, y	self-defined functions
z	the position of particle
**Greek symbols**	
γ	friction coefficient
η	efficiency
μ	asymmetry of the potential
ω, τ	temperature ratio
β	dimensionless heat leakage coefficient
**Abbreviations**	
CR	cold reservoir
FTT	Finite time thermodynamics
HE	heat engine
HR	hot reservoir
TBHE	thermal Brownian heat engine
VM	viscous medium
